# Dynamic Speed of Sound Adaptive Transmission–Reflection Ultrasound Computed Tomography

**DOI:** 10.3390/s23073701

**Published:** 2023-04-03

**Authors:** Xiangwei Lin, Hongji Shi, Zhenyu Fu, Haoming Lin, Siping Chen, Xin Chen, Mian Chen

**Affiliations:** School of Biomedical Engineering, Shenzhen University, Nanshan District, Shenzhen 518057, China

**Keywords:** ultrasound computed tomography, quantitative speed of sound, simultaneous algebraic reconstruction technique, SoS adaptive DAS beamforming

## Abstract

Ultrasound computed tomography (USCT) can visualize a target with multiple imaging contrasts, which were demonstrated individually previously. Here, to improve the imaging quality, the dynamic speed of sound (SoS) map derived from the transmission USCT will be adapted for the correction of the acoustic speed variation in the reflection USCT. The variable SoS map was firstly restored via the optimized simultaneous algebraic reconstruction technique with the time of flights selected from the transmitted ultrasonic signals. Then, the multi-stencils fast marching method was used to calculate the delay time from each element to the grids in the imaging field of view. Finally, the delay time in conventional constant-speed-assumed delay and sum (DAS) beamforming would be replaced by the practical computed delay time to achieve higher delay accuracy in the reflection USCT. The results from the numerical, phantom, and in vivo experiments show that our approach enables multi-modality imaging, accurate target localization, and precise boundary detection with the full-view fast imaging performance. The proposed method and its implementation are of great value for accurate, fast, and multi-modality USCT imaging, particularly suitable for highly acoustic heterogeneous medium.

## 1. Introduction

Ultrasound computed tomography (USCT) is able to visualize an imaging region of interest with different modalities, including the quantitative spatial distribution of speed of sound (SoS), acoustic attenuation, and pulse–echo reflection images [1,2,3,4,5]. With the opposite transmitted pulse and adjacent received ultrasonic echo, this imaging modality can provide non-ionizing, low-cost, and multiple modalities, compared with other imaging techniques such as X-ray computed tomography (CT), magnetic resonance imaging (MRI), and conventional B-mode ultrasound imaging (US) [6,7,8]. It is an attractive tool for quantitatively imaging targets with highly acoustic heterogeneous properties [9,10,11,12], such as malignant tumors in the breast, metal stents in the vessel, peripheral vascular imaging, small animal whole-body imaging, and industrial nondestructive testing. However, previous studies individually focused on either the quantitative transmission information or the qualitive reflection images of the USCT, and the information provided was isolated [11,13]; thus, it needs to be integrated together to avoid information loss, inaccurate spatial positioning, deformed geometric size, and blurred boundary delineation, especially in the above variable SoS situations. 

Although USCT provides the quantitative estimates of acoustic speed in transmission USCT and the qualitative delineation of the different acoustic impedance in reflection USCT, the inside SoS information and outside boundary structure need to be integrated to obtain a more refined target structure. This demand is prominent in the case of accurate imaging positioning with heterogeneous acoustic speed, such as in breast tumor margin identification, which should be at the sub-millimeter level to assist tumor removal surgery [14]. Mark, A.A., et al. established ultrasound numerical models for virtual imaging trials for breast with acoustic variation [15]. The neural network separately proposed by M. Yuchi et al. [16] and A. Mohamed et al. [17] can achieve image restoration from sparse transmissions of USCT. Refractive ray-tracing approaches [18,19] and the diffraction tomography method [20] were proposed to approximate the refracted, diffracted acoustic field under practical heterogenous acoustic condition. Compared with the classic straight-ray-based filtered back-projection methods [21,22], the simultaneous algebraic reconstruction technique (SART) algorithm [23,24] has the potential to be integrated with the US signals from refracted and diffracted transmission acoustic fields to obtain a more accurate two-dimensional SoS map globally. 

Constant speed is typically set in the delay and sum (DAS) beamforming in the reflection USCT. However, this assumption cannot match the practical acoustic heterogeneous tissue [25], and such constant SoS causes wavefront aberrations, degrading the imaging accuracy. Y. Jing et al. focused on the numerical study of transcranial US imaging with SoS map from CT image [26]. In our previous study, the approximately SoS map was derived from the MRI scans [27]. Both studies used the multi-stencils fast marching (MSFM) method to calculate the two-dimensional delay time map between the coordination of the transmitter–receiver in transducer and imaging pixels, but the registration accuracy between different imaging modalities needs to be improved. The SoS given by R. Ali et al. addressed the axial estimation rather than the two-dimensional map in the imaging plane [28]. Y. Matsumoto et al. computed the SoS in the USCT, and the delay time was also derived by MSFM method [29], whereas the SoS difference was relatively low and the sparse sampling effect was not clarified in its experiment. Current USCT system generally has large data acquisition channels to meet the spatial sampling requirement [12,30]. To reduce the hardware cost and accelerate acquisition speed, less channels are required, and, thus, the sparse sampling effect should be mitigated. 

In this work, the dynamic SoS map derived from the transmission USCT was adapted for the correction of the acoustic speed variation in the reflection USCT. The time of flights (ToFs) from the transmitter–receiver pair were selected via P-phase picker method [31]. This high-quality ToFs tomography can efficiently and reliably produce the acoustic speed map of the target. Dynamic SoS map can not only facilitate diagnosis due to its quantitative sensitivity to the specific acoustic speed in the imaging target but also adapt the reflection imaging structure in acoustic heterogenous medium. The remainder of this paper is organized as follows. In Section 2, the theories on the dynamic SoS adaptive transmission–reflection USCT are provided, including the SART algorithm in transmission USCT, the MSFM method, and full-angle spatial compounding DAS reconstruction in reflection USCT. The numerical model and the simulation results are demonstrated in Section 3. Concerning the difference between the simulation and experiment, the phantom and in vivo experiments are separately conducted in Section 4 with the custom-made ring array ultrasound transducer. Section 5 presents the conclusion of the above framework, where the other possible applications are stated.

## 2. Methods

We briefly introduce the proposed method. The dynamic SoS-corrected transmission–reflection USCT process includes three steps: (1) The dynamic SoS map was firstly restored via an iterative method with the transmitted signals; (2) The MSFM method was used to calculate the delay time from each element to the imaging field of view on the basis of the above variable SoS map; and (3) The delay time in the conventional constant speed assumed full-view DAS spatial compounding reconstruction would be replaced by the practical measured delay time in the reflection USCT. 

Taking into account the situation that acoustic refraction, diffraction, and scattering co-exist in the practical target, such as tumor in the breast or bone in soft tissue, the SART algorithm was employed in the first step. It can update all rays passing through a pixel grid at a certain projection angle in each iteration; hence, the measurement errors and interference factors can be suppressed with improvement of restoration accuracy. The ToFs (*T_φ_*) were selected via the method from ref. [31] within the limited range relative to the geometric distance of individual array elements. In addition, the pixel grid-based iterative algorithm can be realized with GPU, avoiding the traditional time-consuming calculation. The iterative solution of SART given by Andersen, A.H. and Kak, A.C. [24] is,

(1) $$s_{j}^{\left(k+1\right)}=s_{j}^{\left(k\right)}+\lambda \frac{\sum_{t_{i}\in T_{\varphi }}c_{i}}{\sum_{t_{i}\in T_{\varphi }}w_{ij}},c_{i}=\frac{t_{i}-\sum_{n=1}^{N}w_{in}s_{n}^{\left(k\right)}}{\sum_{n=1}^{N}w_{in}^{2}}w_{ij}$$

where *s_j_* is the value of the spatially variable acoustic speed, *k* is the iteration step, *i* is the index of projection ray, *j* is the index of the reconstructed pixel grid (1 ≤ *j* ≤ *N*), *w* is the weight factor that relates the grid (*j* or *n*) to the ToFs at the projection *i*, *λ* is the relaxation factor that controls the convergence of the reconstruction (0 < *λ* ≤ 1), *t_i_* is the *i*-th projection in the set *T* with the certain transmitted channel *φ* in the ring array transducer, and *c_i_* is the weighted error between actual projection data and estimated projection data, which updates at each iteration. After reshape *s_j_*, the two-dimensional SoS map, which can be instantly adjusted according to the practical target that was obtained. 

On the basis of the above measured *s_j_*, the MSFM method further computed the two-dimensional delay time map (*T_m,n_*) between the transmitter–receiver pairs and each grid point (*m*, *n*) in the imaging field of view. It utilized the Eikonal equation to approximate the motion of the acoustic front wave, which can propagate along the orthogonal direction and diagonal direction of the grid. The details of MSFM can be seen in our previous work [27]. In ref. [27], the transducer is a linear array, whereas the transducer here is a ring array, thus the coordination of the transducer element for transmitting and receiving the US pulses needs to be changed accordingly. 

In the conventional reflection mode USCT, the image is restored via DAS beamforming based on constant assumed sound speed. The weighted DAS reconstruction method with full-angle spatial compounding function is,

(2) $$R_{DAS}(m,n)=\sum_{a=1}^{A}\sum_{k=1}^{K}R(k,t-\Delta t_{m,n})W_{m,n}$$

where *R_DAS_(m,n)* is the beamformed US intensities at the pixel grid (*m*, *n*), and *R(k*,*t)* is the raw channel data that are acquired by the transducer channel *k* at the sampling time *t*. *K* denotes the total number of adjacent transducer channels involved in the reflection (1 ≤ *k* ≤ *K*). *A* denotes the total sampling angles that are determined by the number of the ring array transducer (1 ≤ *a* ≤ *A*). Δ*t_m_*_,*n*_ is the time delay from each element of transducer channel *k* to the predefined imaging grid (*m*,*n*), which, in most cases, is computed with constant speed. *W_m_*_,*n*_ is the apodization factor that reduces the cumulative intensity of beamformed US signals, which cannot be detected due to the limited reception angle of the transducer element and its corresponding acoustic field. In this study, this acoustic field can be simulated via acoustic field model and determined by practically measuring the intensities of the oppositely received signals propagated in water (Section 4.1). With the delay time map (*T_m_*_,*n*_) calculated in the second step, the SoS adaptive DAS beamforming is

(3) $$R_{SoS+DAS}(m,n)=\sum_{a=1}^{A}\sum_{k=1}^{K}R(k,t-T_{m,n})W_{m,n}$$

where constant speed calculated delay map Δ*t_m_*_,*n*_ is replaced by the MSFM method calculated delay map *T_m_*_,*n*_. It can be derived dynamically from the variable SoS map. With the SoS correction, calibration of the reconstructed reflection images is not necessary, which can be verified in the following simulations and experiments. 

## 3. Simulation and Results

### 3.1. Simulation Models

**Figure 1:**
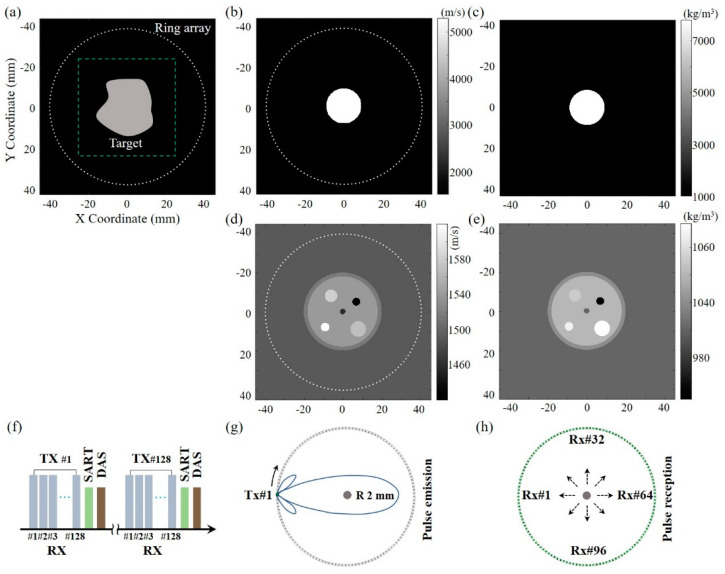
Simulation configuration of the ring array USCT. (**a**) Simulation setup. The medium speed (**b**) and density (**c**) of circular steel target. The medium speed (**d**) and density (**e**) of multi-layer targets. (**f**) Imaging sequence. Pulse emission (**g**) and reception (**h**) schematic in the first cycle.

The simulation models were established to validate that the multi-modality capabilities and precise imaging positioning can be realized via the dynamic SoS-corrected DAS reconstruction in the transmission–reflection USCT. The k-Wave toolbox was chosen for the transmitting and receiving of ultrasound signals, and the simulation configuration is shown in Figure 1a, where the imaging targets were placed in the center area (green dotted box) of the ring array transducer. Considering the practical tissue condition, two types of numerical models were set to highlight the feasibility and advantage of the proposed method. One is a circular target (18.00 mm in diameter) to simulate tissue with highly different acoustic speed, and its acoustic speed and density are labelled in Figure 1b and Figure 1c, respectively. It worth noting that this model was designed with the dominant acoustic refraction (the interface between water and steel) and large SoS variation (5300 m/s in steel and 1490 m/s in coupling water), simulating situations such as puncture with a metallic needle and soft tissue containing bone. 

The other model had relatively complicated multi-targets to mimic the multi-layer structures in practical biological tissue, as shown in Figure 1d,e. The imaging sequence for the transmission–reflection USCT is shown in Figure 1f. The pulse was transmitted from first element (TX#1, Figure 1g), and all the channels (RX#1–#128, Figure 1h) received the opposite transmitted and adjacent reflected signals afterwards. The active transmit pulse was subsequently moved by one channel, and this pulse emission–reception cycle was repeated for all the left channels until the last 128th element. Note that the imaging sequence is the same in the following experimental study.

### 3.2. Simulation Results

**Figure 2:**
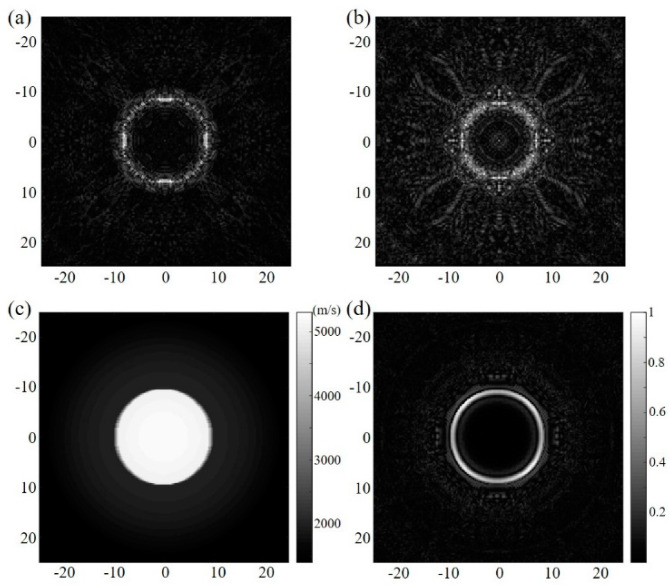
Simulation results. The reflection image from the constant acoustic speed (**a**) 1490 m/s and (**b**) 1540 m/s. (**c**) SoS map derived from the transmission USCT. (**d**) SoS adaptive reflection image in USCT.

Figure 2a,b separately show the reflection images of the circular steel target reconstructed via the constant sound speed of 1490 and 1540 m/s. These acoustic speed values were chosen according to the average sound velocity on the acoustic path, including the inner structure and the surrounding coupling water. The measured diameters are, separately, 16.80 and 14.60 mm, which are obviously different from the simulation settings. In addition, the image contrast changes with acoustic speed. Therefore, the boundary and the size of the target cannot be accurately obtained. With the ToFs selected in the transmission USCT, the SoS map can be derived, as shown in Figure 2c. The SoS adaptive DAS reconstructed image can be seen in Figure 2d. Comparing these results, the size, shape, and overall contrast of the imaging target are shown to be improved, with no additional calibration to select the optimal constant acoustic speed. Even if the sound speed difference is greater than 255% (from 1490 m/s in surrounding medium to 5300 m/s in the imaging steel), the proposed method still works, which is important for precise imaging in a variable acoustic speed situation.

**Figure 3:**
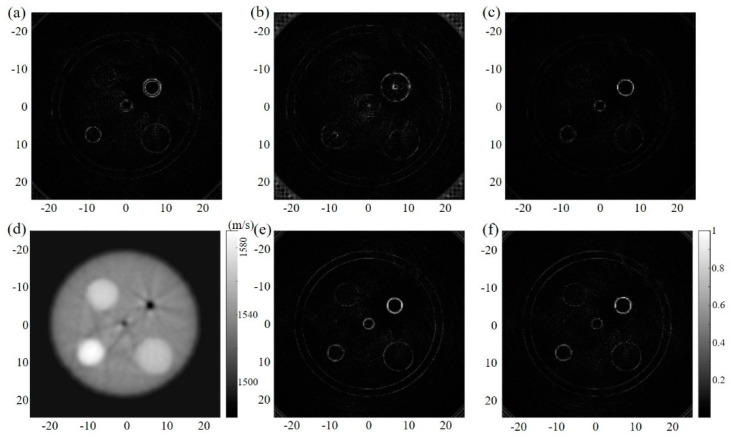
Simulation results. The reflection image from the constant acoustic speed (**a**) 1490 m/s, (**b**) 1540 m/s, and (**c**) 1505 m/s. SoS map derived from the transmission USCT (**d**) and corresponding reflection image (**f**). (**e**) Reflection image in USCT adapted with the preset SoS map.

To further verify the feasibility, a relatively complex multi-target numerical model was established to study the influence of various acoustic speed differences. The acoustic speeds to recover the reflection images of Figure 3a,b are 1490 and 1540 m/s, respectively, whereas the acoustic speed is 1505 m/s to obtain Figure 3c. As the speed iterates from 1420 m/s to 1620 m/s with the step size 5 m/s, the optimized speed is selected according to the evaluation criterion that the target size in the reconstructed image can match the size in Figure 1d. Figure 3d shows the reconstructed SoS map, which is similar to the gold standard shown in Figure 1d. Figure 3f is the reflection USCT-reconstructed result corresponding to the SoS map in Figure 3d, and the corrected reconstruction result corresponding to the SoS map in Figure 1d is shown in Figure 3e. Compared with Figure 3e,f, it can be observed that the reconstruction results (including the target locations, sizes, and boundary shapes) are almost the same, which proves that the dynamic SoS distribution can not only provide the inside quantitative information but also compensate for the reflection image in the reflection USCT. It is worth noting that the reflection results (Figure 3e,f) can directly reflect the structural information, whereas the results from the constant speed restoration need further calibration to search for the desired acoustic speed. 

## 4. Experiments and Results

### 4.1. Experiment Setup

**Figure 4:**
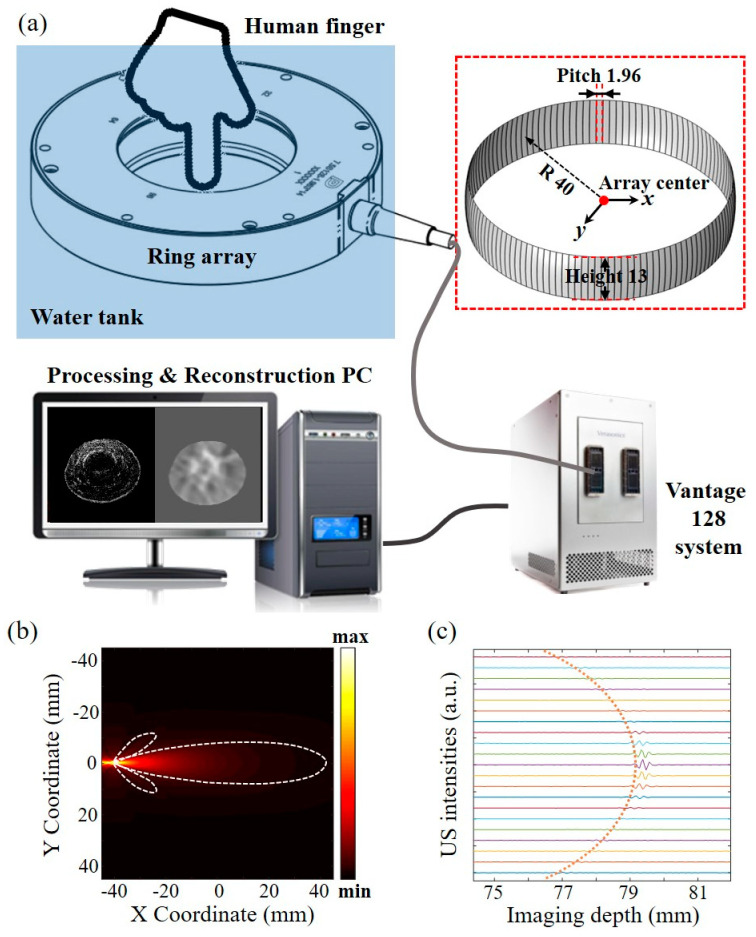
Schematic of the experimental setup and system calibration. (**a**) Experimental setup. (**b**) Acoustic field simulation of the first transmit. (**c**) US intensities versus the imaging depth.

The schematic of the ring-array-based transmission and reflection USCT experimental setup is shown in Figure 4a. The setup mainly includes a custom-made ring array ultrasound transducer, a multi-channel ultrasound data transceiver platform (Vantage128, Verasonics, Kirkland, WA, USA), and the GPU-based signal processing and image reconstruction personal computer (PC). As shown in the inset, the ring array transducer has 128 elements, 40 mm radius, 13 mm height, and 1.96 mm pitch. Here the element spacing is 0.1 mm, which can maximize the reception angle of the ring array element. Each element has a central frequency of 7.5 MHz with 90% bandwidth. The US signal was amplified at the same level by the amplifier that comes with Vantage system at the level of 30 dB, 31.25 MHz sampling rate, and 14 bits resolution. A steel bar and human finger were inserted in the coupling water as the imaging targets. Each transducer element was excited with a 20 V_pp_ positive pulse. The imaging sequence is the same with the simulation setting, where the first transmitting pulse was fired and then all the channels (including the transmitting channel) received the response after a certain propagation time. Here, 128 transmitting and receiving events took place to acquire the full-view US data, and the entire data acquisition time was 25.6 ms. 

After data acquisition, the channel data was processed on a computer (Inter Core i7@3.60 GHz, 32 GB of RAM, NVIDIA GeForce RTX 3060TI) with the proposed method in Section 2 sequentially. The SART reconstruction, MSFM-based delay map calculation, and the weighted DAS reconstruction were implemented on GPU using MATLAB to achieve high-speed processing. Thus, the data processing above can be completed in one data acquisition cycle, enabling fast imaging capacity. The acoustic beam in the elevation was focused at 36 mm due to the concave cylindrically focused element, providing a uniform imaging field of view 20 × 20 mm^2^. In order to accurately characterize the receiving angle of the transducer element, simulation and practical tests were performed separately. The simulated transmitting acoustic field from the first element is shown in Figure 4b, in which the reception angle is approximately 30 degrees according to the acoustic reciprocal theorem [32]. By measuring the intensities of the oppositely received signals propagated in pure water, the reception angle was consistent with the above ultrasound field analysis results, as depicted by the curved dashed line in Figure 4c. This angle is relatively large, thereby mitigating the effect of sparse sampling. Compared with the directional reception in the simulation setup, this angle was restricted in the hardware, and, thus, it should be integrated in the *w_ij_* and *W_m,n_*. In addition, the number of adjacent channels *k* around the transmit channel should also be adjusted according to the imaging quality.

### 4.2. Phantom Experimental Results

**Figure 5:**
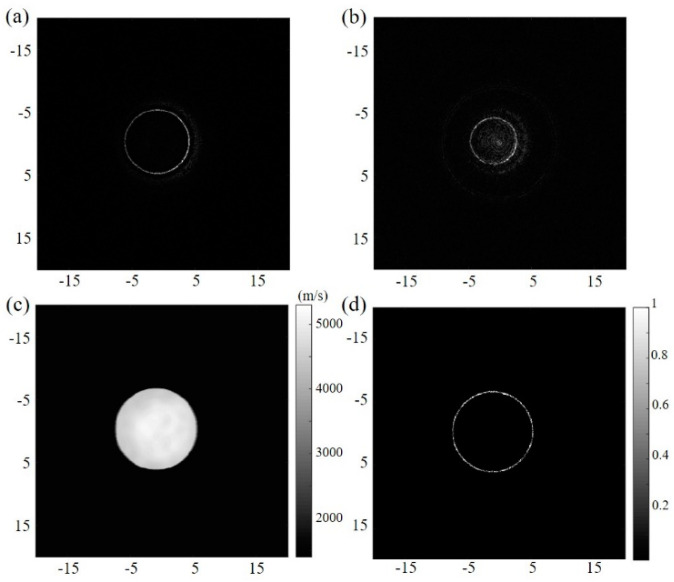
Phantom experimental results. The reflection image from the constant acoustic speed (**a**) 1490 m/s and (**b**) 1540 m/s. (**c**) SoS map derived from the transmission USCT. (**d**) SoS adaptive reflection image in USCT.

To verify the practical feasibility of the proposed method, a steel bar (diameter of 12.7 mm) with relatively large acoustic speed difference was placed in the central area of ring array ultrasound transducer. The reconstructed results shown in Figure 5a and b represent constant acoustic speed 1490 and 1540 m/s, respectively, following the ultrasonic pulse emission and reception cycle. Similar to the results from the numerical model, the ring shape of the phantom can be rendered, but the sizes (10.22 versus 7.41 mm) are seriously deteriorated due to the variation of the assumed constant speed. Hence, the calibration still needs to be additionally performed. With the two-dimensional SoS map, i.e., Figure 5c,d, is the SoS adaptive reflection image, from which we can measure that the position, shape, and size are similar to the real target. The contrast-to-noise ratio (CNR) is used to characterize the restored images and can be defined as $20\log_{10}(({\bar{I}}_{t}-{\bar{I}}_{b})/\sqrt{\sigma_{t}^{2}+\sigma_{b}^{2}})$, where ${\bar{I}}_{t}$, ${\bar{I}}_{b}$, $\sigma_{t}^{}$, and $\sigma_{b}^{}$ are the mean intensities of the target region, background region, and the standard deviations of the above corresponding regions, respectively. The target region is selected at the edge of the steel bar, and the background region was selected in the upper left corner of each figure. The measured CNRs for Figure 5a,b,d are 12.55, 12.15, and 17.66 dB, separately. In addition, the SoS map can provide not only the target inside structure but also the acoustic speed property, where multiple contrast can be obtained in the same platform.

### 4.3. In Vivo Experimental Results

**Figure 6:**
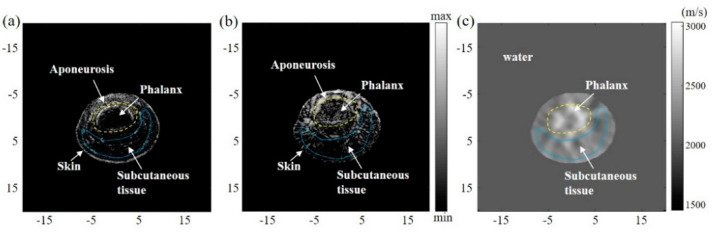
In vivo experimental results. (**a**) The reflection image from the constant acoustic speed of 1495 m/s. (**b**) SoS adaptive reflection image in USCT. (**c**) SoS map derived from the transmission USCT.

Preliminary research was further carried out on a human finger, which is in compliance with the research committee of Shenzhen University. The index finger of the volunteer was immersed in the water, and the imaging plane laid in between the proximal interphalangeal and the distal interphalangeal. The constant speed (1495 m/s) reconstructed reflection image is shown in Figure 6a, and this speed was calibrated with the iteration method (from 1450 to 1600 m/s with the step size of 5 m/s) to guarantee the real finger size. Figure 6c shows the reconstructed SoS map from the SART method using Formula (1), and we can see the phalanx area clearly. It should be noted that the image contrast is relatively low due to the fact that the acoustic speed difference in tissue is relatively small. Figure 6b shows the SoS-corrected reflection image. Compared with Figure 6a, the tissue structure, especially the phalanges and subcutaneous tissue (labelled by the yellow and blue dashed line), can be observed in both Figure 6b,c with two different acoustic properties. The measured CNRs for Figure 6a,b are 20.58 and 23.01 dB, separately. The target region was selected in the boundary of the phalanx. Note that one mask selecting the FOVs was created and blurred with a three-pixel standard deviation Gaussian filter. The blurred mask images were then multiplied with the three reconstructed images to produce the final restored image.

## 5. Conclusions

Our method and its implementation can promote the ultrasound image quality in the reflection USCT with the SoS information derived from the transmission USCT. Beyond the multi-contrast imaging ability (inside quantitative acoustic speed and outside acoustic impedance difference), this approach can improve the accuracy of target positioning and boundary delineation with fast full-view imaging ability. The numerical simulations were conducted firstly to validate our assumption. Then, the results from the phantom and in vivo experiments further verified that the proposed method can be applied in the highly acoustic speed heterogeneous medium. With the custom-made ring array transducer, the large reception angle can mitigate the effect of sparse sampling with limited element number of the ring array. The restored target information can be more accurately acquired for the SoS-corrected reflection US images without additional calibration to search for the optimized constant speed. The imaging sequence with all transmitting–receiving array elements can be accelerated by the GPU, enabling full-view fast imaging capacity. Therefore, our approach and the corresponding system enable fast, accurate, and multi-modality imaging.

In future work, the proposed method and system have the potential to be applied to biological tissues with large difference in acoustic speed, such as transcranial ultrasound neuromodulation and whole-body imaging of small animals. Meanwhile, the image fusion technique will be applied to co-register the SoS map, acoustic attenuation, and boundary structure. Thus, the inside acoustic speed and reflectional acoustic impedance in the boundary may be integrated. In addition, even if the reception angle is maximized in our ring array transducer, the sparse sampling effects still exist theoretically, and the compressed sensing method will be used to further conquer this effect with improvement of imaging quality. Moreover, based on the same hardware platform, the dynamic SoS map from the USCT will be incorporated in photoacoustic-computed tomography to provide additional optical contrast to reveal multiple physicochemical mechanisms.

## Data Availability

Not applicable.
